# Antimicrobial and Cytotoxic Activity of Endophytic Fungi from *Lagopsis supina*

**DOI:** 10.4014/jmb.2211.11055

**Published:** 2022-12-30

**Authors:** Dekui Zhang, Weijian Sun, Wenjie Xu, Changbo Ji, Yang Zhou, Jingyi Sun, Yutong Tian, Yanling Li, Fengchun Zhao, Yuan Tian

**Affiliations:** 1 Department of Microbiology, College of Life Science, Key Laboratory for Agriculture Microbiology, Shandong Agricultural University, Taian 271018, P.R. China; 2 College of Life Science, Shandong First Medical University & Shandong Academy of Medical Sciences, Taian 271016, P.R. China

**Keywords:** Endophytic fungi, *Lagopsis supina*, antimicrobial activity, cytotoxic activity

## Abstract

In this study, five endophytic fungi belonging to the *Aspergillus* and *Alternaria* genera were isolated from *Lagopsis supina*. The antimicrobial activity of all fungal fermented extracts against *Staphylococcus* and *Fusarium graminearum* was tested using the cup-plate method. Among them, *Aspergillus ochraceus* XZC-1 showed the best activity and was subsequently selected for large-scale fermentation and bioactivity-directed separation of the secondary metabolites. Four compounds, including 2-methoxy-6-methyl-1,4-benzoquinone (1), 3,5-dihydroxytoluene (2), oleic acid (3), and penicillic acid (4) were discovered. Here, compounds **1** and **4** displayed anti-fungal activity against *F. graminearum*, *F. oxysporum*, *F. moniliforme*, *F. stratum*, *Botrytis cinerea*, *Magnaporthe oryzae*, and *Verticillium dahliae* with diverse MIC values (128–512 μg/ml), which were close to that of the positive control antifungal, actidione (64–128 μg/ml). Additionally, compounds **1** and **4** also exhibited moderate antibacterial activity against *S. aureus*, *Listeria monocytogenes*, *Escherichia coli*, and *Salmonella enterica*, with low MIC values (8–64 μg/ml). Moreover, compounds **1** and **4** displayed selective cytotoxicity against cancer cell lines as compared with the normal fibroblast cells. Therefore, this study proposes that the endophytic fungi from *L. supina* can potentially produce bioactive molecules to be used as lead compounds in drugs or agricultural antibiotics.

## Introduction 

Endophytes are characterized as microorganisms residing in healthy plants for part of their life cycle without causing any noticeable disease symptoms in their hosts [1]. To adapt to the internal environment of their hosts, they usually have unique physiological and metabolic mechanisms, which increase the possibility of producing new compounds [2]. The search for natural bioactive products from endophytic fungi has markedly increased over the last few years [3,4]. Molecules with antimicrobial, anti-tumor, anti-inflammatory, and anti-oxidative activities belonging to various structural types, including terpenoids, steroids, alkaloids, flavonoids, and phenols, have reportedly been isolated from plant endophytic fungal cultures [4-7]. The discovery of medicinally important lead compounds, including taxol, camptothecin, and vinca alkaloids from endophytic fungi has paved the way for exploring bioactive metabolites for commercial usage [8].

*Lagopsis supina*, also known as “Xiazhicao,” is a traditional Chinese medicinal plant for the treatment of blood stasis syndrome. Phytochemical studies of *L. supina* have discovered diverse molecules, including flavonoids [9], glycosides [10], and diterpenoids [11]. Modern pharmacological studies have shown that *L. supina* displayed diverse biological effects, including improvement of blood and lymph microcirculation [12] as well as myocardioprotective [13], anti-inflammatory [11], and antioxidant activity [14]. However, to the best of our knowledge, the bioactive molecules of endophytes from *L. supina* have not been reported to date.

Therefore, in this work, we isolated and studied endophytic fungi from *L. supina*, and the *Aspergillus ochraceus* XZC-1 strain exhibited the best anti-fungal and antibacterial activity. Chemical investigation of the strain resulted in obtaining four compounds, among which two compounds showed antimicrobial and cytotoxic activities. The findings presented here provide the scientific basis for exploiting endophytes from *L. supina* as biological sources of antimicrobial and cytotoxic compounds.

## Materials and Methods

### Pathogens and Media

Four pathogenic bacteria, including *Staphylococcus aureus* (ATCC25923), *Listeria monocytogenes* (CGMCC1.10753), *Escherichia coli* (ATCC 8739), and *Salmonella enteritidis* (ATCC13076) were purchased from the Shanghai Bioresource Collection Center (SHBCC). Seven pathogenic fungi, including *Fusarium graminearum*, *F. oxysporum*, *F. moniliforme*, *F. stratum*, *Botrytis cinerea*, *Magnaporthe oryzae*, and *Verticillium dahliae*, were all separated and identified by our laboratory [15,16].

PDA (potato dextrose agar) medium [20 g potato, 2 g dextrose, 1.5 g agar, and 100 ml deionized water], ME (malt extract) medium [2 g raw malt, 2 g sucrose, 0.1 g peptone, and 100 ml deionized water], and LB (Luria-Bertani) medium [1 g tryptone, 0.5 g yeast extract, 1 g NaCl, 100 mL deionized water, and pH 7.2] were used in this study.

### Isolation of Endophytic Fungi

Healthy *L. supina* was collected in Tai’an, Shandong Province, China. Endophytic fungi were isolated according to methods detailed previously [15]. The tissues were washed with distilled water and sterilized as follows: First, tissue samples were soaked in 0.05% Tween 20 for 40 s, 5% hypochlorite for 3 min, 2.5% thiosulfate for 5 min, and 75% ethanol for 2 min, and finally rinsed with sterile distilled water for 5 min. Subsequently, samples were cut into pieces (1 cm^2^ of the leaf and 1 cm length of the stem) and placed onto the PDA medium with 100 μg/ml ampicillin. These samples were incubated aerobically for five days at 28°C. The pure colonies were transferred to fresh PDA medium and preserved at 4°C for further use.

### Identification of Endophytic Fungi

The endophytic fungi were identified using morphology and molecular biology. For morphological identification, endophytic fungal colonies, including the hypha and conidial head, were observed after 5 days of incubation on PDA at 28°C in the dark. For molecular biological identification, fungal genomic DNA was first extracted with the Fungal DNA Kit (Omega Bio-tek, Inc., USA) according to the manufacturer’s recommendations. The first internal transcribed spacer (ITS-1) of ribosomal DNA (rDNA) of the strains was amplified and sequenced by Ruibio Bio Tech Co., Ltd. (China). Subsequently, the ITS gene sequences were subjected to a BLAST search in the GenBank database. The closely related strains were obtained to establish a neighbor-joining distance tree using the MEGA 7.0 software, with 1,000 replicates being used for the bootstrap analysis [17].

### Fermentation and Primary Screening of the Endophytic Fungi

The fungal mycelia of each isolate were inoculated into a 1-L Erlenmeyer flask containing 400 ml of ME medium, and cultured for 10 days at 28°C on a rotary shaker (180 rpm). The culture was filtered through four layers of muslin cloth [18]. The resulting filtrate was subsequently added with an equal volume of ethyl acetate and extracted twice. Finally, the extracted solution was concentrated by rotary evaporation and re-dissolved with methanol to obtain the crude extract solution (20 mg/ml), which was used for further bioactivity testing.

The anti-fungal and antibacterial activities were tested using the cup-plate method [15, 16]. First, the LB agar containing 10^7^ CFU/ml of *S. aureus* or PDA mixed with 10^7^ CFU/ml spores of *F. graminearum* was poured into a dish (Oxford cups pre-placed equidistantly). After solidification, wells were bored into the medium, and 100 μl of crude extract solution (20 mg/ml) was poured into each well. Methanol was used as the negative control. The plates containing bacteria were cultured at 37°C for 24 h, while those containing fungi were cultured at 28°C for 3 days. Finally, the diameters of the inhibition zones were measured.

### Large-Scale Fermentation and Antimicrobial Activity Verification

Upscale fermentation was performed in a fermenter (100 L) containing 50 L of ME broth. After 10 days of cultivation at 28°C, the fermentation broth was filtered and extracted with ethyl acetate. The solvent was removed via rotary evaporation to yield the crude extract.

Anti-fungal and antibacterial activities of the crude extracts from large-scale fermentation were verified. The anti-fungal activity against five phytopathogens (*F. graminearum*, *F. oxysporum*, *F. moniliforme*, *F. stratum*, and *B. cinerea*), was tested using the mycelial growth rate method [19]. First, 20 ml of pre-heated PDA was added along with 100 μl of 20 mg/ml crude extract (experimental group) or methanol (control group). The mixture was mixed well and poured into a Petri dish. After solidification, a pathogenic fungal cake (0.7 cm) was placed at the center of the prepared dish and incubated for 6 days at 28°C. To calculate the growth inhibition rate of the crude extract, the diameter of the pathogenic fungus colony was determined using the cross method. The inhibition rate was calculated according to the following formula: (1−D_a_/D_b_)×100%, where D_a_ and D_b_ are the colony diameters in the experimental and control plates. Three replicates were used for each anti-fungal activity test. The antibacterial activity of the crude extract was tested against four pathogens, *S. aureus*, *L. monocytogenes*, *E. coli*, and *S. enteritidis*, using the abovementioned cup-plate method.

### Purification and Characterization of Compounds

The crude extract was added to the silica column chromatography and eluted with petroleum ether/ethyl acetate mixtures of increasing polarity (50:1, 20:1, 10:1, 5:1, 2:1, 1:1; v/v) to obtain six fractions (F1–F6). Then, the fractions that showed antimicrobial activity were purified on a Sephadex LH-20 and semipreparative reversed-phase HPLC to extract the bioactive molecules.

The structures of the separated compounds were identified using a nuclear magnetic resonance (NMR) spectrometer (Bruker Avance 400, Bruker BioSpin AG, Switzerland). ^1^H-NMR and ^13^C-NMR spectra were measured in CDCl_3_ or acetone-*d_6_* using tetramethylsilane (TMS) as the internal standard.

### Anti-Fungal Activity of the Compounds

Seven plant pathogens (*F. graminearum*, *F. oxysporum*, *F. moniliforme*, *F. stratum*, *B. cinerea*, *M. oryzae*, and *V. dahliae*) were used for measuring the anti-fungal activity. According to the methods described previously [15], first, 90 μl of fungal spores solution (1~5 × 10^5^ CFU/ml) along with 10 μl of the compounds (16–512 μg/ml) were added to each well of a 96-well plate. The plate was then incubated at 28°C for 48 h. Finally, the absorbance was measured at 600 nm. The minimum inhibitory concentration (MIC) of the compound was determined with the absorbance value close to the blank value with no obvious turbidity in the pores. Actidione was used as the positive control. Three replicates were carried out for each anti-fungal activity test.

### Anti-Bacterial Activity of the Compounds

MIC values were determined as described previously [16]. In brief, four pathogenic bacteria (*S. aureus*, *L. monocytogenes*, *E. coli*, and *S. enteritidis*) were added to a series of media containing a gradient concentration of the obtained compounds and incubated for 24 h at 37°C on a rotary shaker at 200 rpm. The final concentrations of the compounds used in the media were 128, 64, 32, 16, 8, 4, 2, and 1 μg/ml. LB broth containing ampicillin and that without any compounds were used as the positive and negative controls, respectively. The final results were representative of three individual experiments. The MIC value was determined as the lowest concentration that could inhibit the growth of the tested microorganism.

### Cytotoxicity of the Compounds

The cytotoxicities of the pure compounds against human lung carcinoma cells A549, hepatoma cells HepG2, and normal human primary fibroblast cells WI-38 were carried out using the MTT colorimetric assay with doxorubicin hydrochloride as the positive control and 0.5% DMSO as negative control [20]. First, 100 μl/well of cells (at a density of 4 × 10^4^ cells/ml) were seeded into 96-well plates and incubated at 37°C for 20 h. Then, the compounds were added into the cell cultures at different concentrations (0.075–1.2 μg/ml of compound **1** and 3.125–50 μg/ml of compound **4**). After 48 h, 20 μl MTT reagent (5 mg/ml), which was dissolved in phosphate-buffered saline (PBS) (pH 7.2), was added to the cells and incubated at 37°C for an additional 4 h. Then, post media removal, 150 μl DMSO (Aladdin Biotech, China) was added into each well and the plate was shaken for 10 min to dissolve the generated formazan crystals. Finally, the absorbance was recorded at 570 nm using a microplate reader (BMG LABTECH, Germany). The 50% inhibition concentration (IC_50_) values against the tumor cells were calculated from a four-parameter logistic equation of the S-type curve using the OriginPro 9.1 software (OriginLab Corporation, USA).

## Results

### Isolation and Identification of Endophytic Fungi

**Fig. 1:**
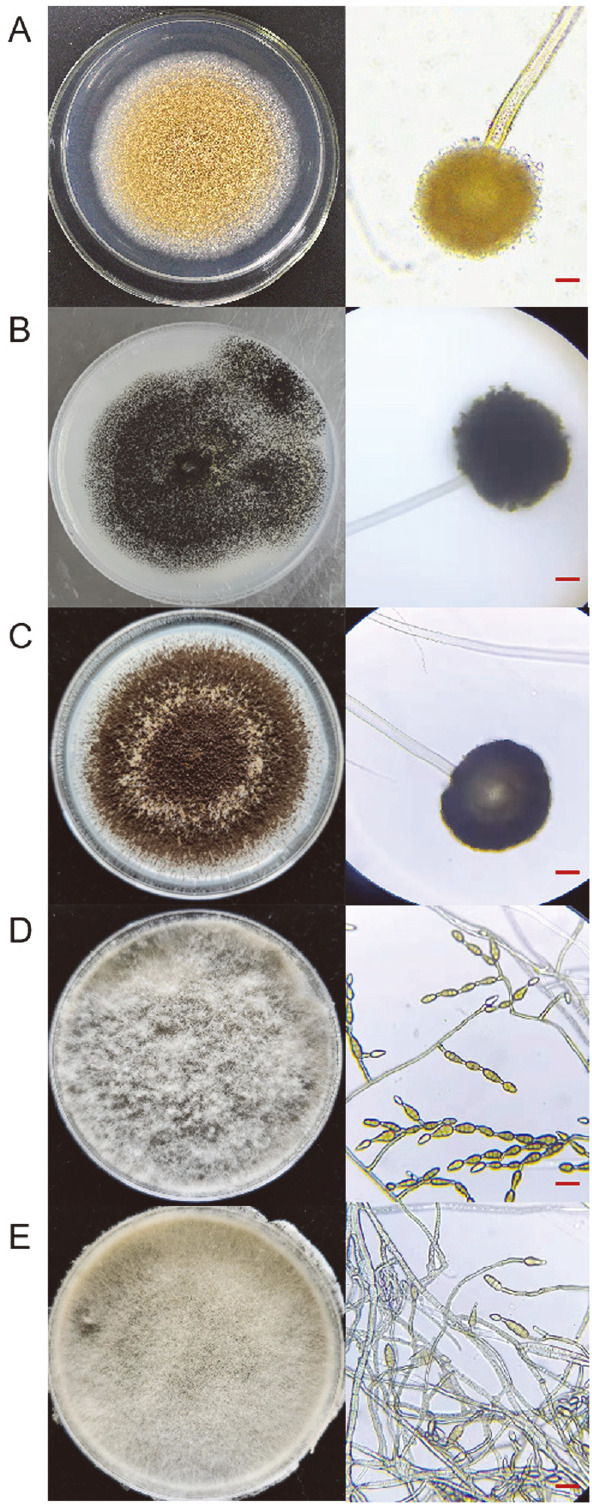
Colonies (left) and microscopic morphology (right) of endophytic fungi from *L. supina*. **A**. XZC-1, **B**. XZC-2, **C**. XZC-3, **D**. XZC-4, and **E**. XZC-5. The scale bar represents 10 μm.

Five endophytic fungi were isolated from *L. supina*, among which the strains (XZC-1 and XZC-2) were obtained from the leaves, while the other three (XZC-3, XZC-4, and XZC-5) were derived from the stems. As shown in Fig. 1, strain XZC-1 had a yellow sporophore with a knob-like top, whereas XZC-2 and XZC-3 showed dark sporophores with knob-like tops, which were all consistent with the morphological characteristics of *Aspergillus*. However, the XZC-4 and XZC-5 strains were found to have brown conidiophores which were obclavate to subcylindrical with transverse and longitudinal septa, thereby indicating that they belonged to the genus *Alternaria*.

**Fig. 2:**
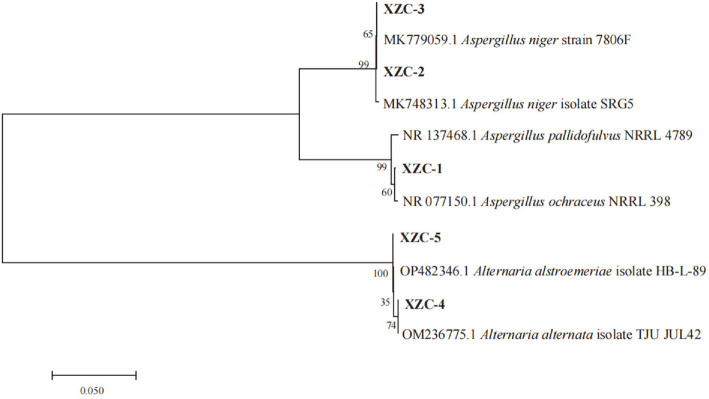
Neighbor-joining phylogenetic tree based on the ITS sequences. The endophytic strains are highlighted in bold. Numbers at the branch points are the bootstrap values based on 1000 replicates. The scale bar represents 0.005 nucleotide changes per position.

The ITS genes of the five fungi were amplified and sequenced (GenBank accession no. OM131592, OP895683–OP895686). As evident from the neighbor-joining tree (Fig. 2), the species most closely related to the XZC-1 strain was *Aspergillus ochraceus* NRRL 398 (NR 077150.1) with 99.63% similarity. Thus, this strain was identified as *A. ochraceus* XZC-1. Similarly, the XZC-2 and XZC-3 strains were identified as *Aspergillus niger*, while strains XZC-4 and XZC-5 were identified as *Alternaria alternata* and *Alternaria alstroemeriae*, respectively.

### Activity Screening of Crude Extracts from Endophytic Fungi

The fermented crude extracts of the five fungi isolated from *L. supina* were tested against the pathogenic bacterium *S. aureus* and the phytopathogenic fungus *F. graminearum*. As shown in Table 1, all the strains inhibited the two tested pathogens. Among them, *A. ochraceus* XZC-1 exhibited the most effective antimicrobial activity and was therefore selected for large-scale fermentation.

### Activity Validation of Crude Extract from *A. ochraceus* XZC-1

**Fig. 3:**
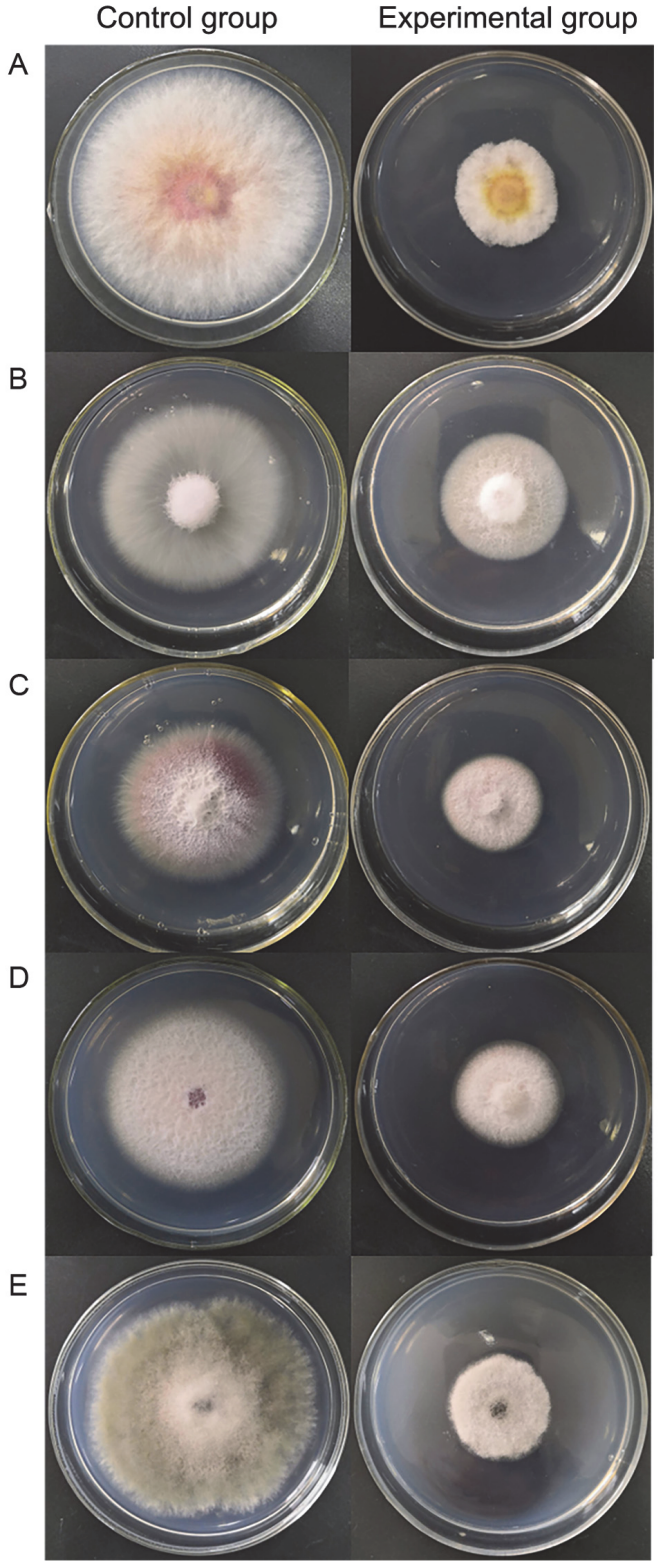
Anti-fungal activity of the crude extract from strain XZC-1 against the five pathogenic fungi. **A**. *Fusarium graminearum*, **B**. *F. oxysporum*, **C**. *F. moniliforme*, **D**. *F. stratum*, and **E**. *Botrytis cinerea*.

After large-scale fermentation, a 12.4 g crude extract was obtained. The crude extract was subjected to an antimicrobial assay to verify its antimicrobial activity. As shown in Fig. 3 and Table 2, the fermented crude extract of XZC-1 showed significant inhibition rates against the five phytopathogenic fungi (61.2% for *F. graminearum*, 32.7% for *F. oxysporum*, 42.3% for *F. moniliforme*, 44.6% for *F. stratum*, and 55.3% for *B. cinerea*).

Table 2 also demonstrated the potent antibacterial activities of the crude extract of XZC-1 with inhibition zones for the four kinds of pathogenic bacteria (34 mm for *S. aureus*, 33 mm for *L. monocytogenes*, 28 mm for *E. coli*, and 32 mm for *S. enteritidis*).

### Structural Characterization of Bioactive Compounds

**Fig. 4:**
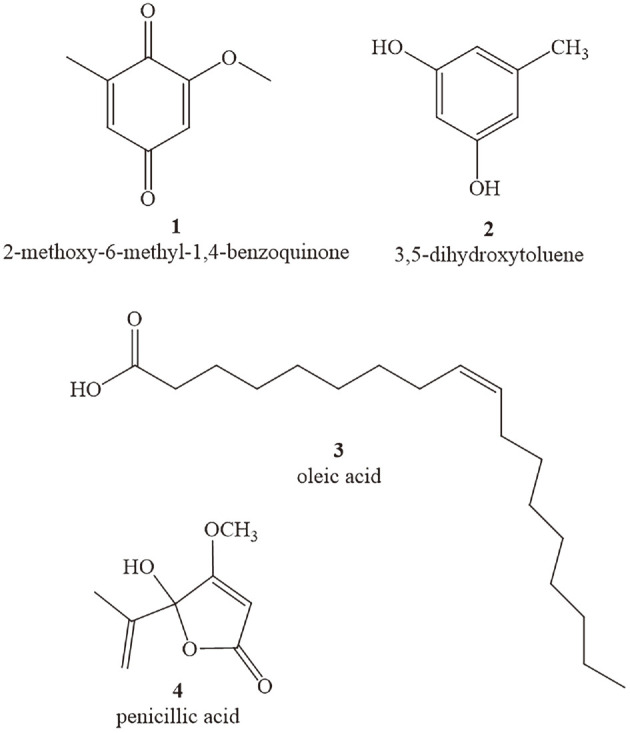
Structures of compounds 1-4.

Four compounds were obtained via bioactivity-guided isolation. The spectral data reported by Chow *et al*. [21] (for compound **1**), Ouyang *et al*. [22] (for compound **2**), Leggio *et al*. [23] (for compound **3**), and Kimura *et al*. [24](for compound **4**) helped identify 2-methoxy-6-methyl-1,4-benzoquinone (**1**), 3,5-dihydroxytoluene (**2**), oleic acid (**3**), and penicillic acid (**4**) (Fig. 4).

Compound **1**: ^1^H-NMR (400 MHz, CDCl_3_) δ = 2.069 (d, *J* = 0.8 Hz, 3H), 3.817 (s, 3H), 5.879 (d, *J* = 1.6 Hz, ^1^H), 6.534-6.546 (m, ^1^H) ppm. ^13^C-NMR (100 MHz, CDCl_3_) δ = 15.52, 56.29, 107.32, 133.86, 143.66, 158.81, 182.40, 187.46 ppm.

Compound **2**: ^1^H-NMR (400 MHz, Acetone-*d_6_*) δ = 2.155 (s, 3H), 6.160 (s, 3H), 8.018 (s, 2H) ppm. ^13^C-NMR (100 MHz, Acetone-*d_6_*) δ = 21.51, 100.65, 108.34 (2C), 140.63, 159.31 (2C) ppm.

Compound **3**: ^1^H-NMR (400 MHz, CDCl_3_) δ = 0.868 (t, *J* = 4.8 Hz, 3H), 1.255-1.342 (m, 22H), 1.610 (dt, *J* = 9.2, 4.8 Hz, 2H), 1.996 (dt, *J* = 8.0, 4.4 Hz, 2H), 2.335 (t, *J* = 4.8 Hz, 2H), 5.315 (dq, *J* = 13.2, 4.8 Hz, 2H) ppm. ^13^C-NMR (100 MHz, CDCl_3_) δ = 14.12, 22.69, 24.69, 27.16, 27.22, 29.04, 29.07, 29.14, 29.33, 29.37, 29.44, 29.53, 29.68, 31.91, 33.80, 129.74, 130.04, 178.66 ppm.

Compound **4**: ^1^H-NMR (400 MHz, CDCl_3_) δ = 1.778 (s, 3H), 3.914 (s, 3H), 5.131 (s, ^1^H), 5.224 (s, ^1^H), 5.491 (s, ^1^H) ppm. ^13^C-NMR (100 MHz, CDCl_3_) δ = 17.38, 59.83, 89.39, 103.03, 116.57, 139.65, 171.16, 179.11 ppm.

### Anti-Fungal Activity of Bioactive Compounds

As reported in Table 3, all seven phytopathogenic fungi were inhibited by compounds **1** and **4**. The MIC values of compounds **1** and **4** were ranged between 128 and 512 μg/ml, which were close to that of the positive control, actidione (64–128 μg/ml). However, compounds **2** and **3** showed no antifungal activity against the seven phytopathogenic fungi (data not shown).

### Antibacterial Activity of the Bioactive Compounds

Compounds **1** and **4** displayed effective antibacterial activity against the four bacterial pathogens. As shown in Table 4, compound **1** exhibited the following MIC values: *S. aureus* (8 μg/ml), *E. coli* (64 μg/ml), and *L. monocytogenes* and *S. enteritidis* (16 μg/ml). However, those for compound **4** were 32 μg/ml for *S. aureus* and 64 μg/ml for *E. coli*, *L. monocytogenes* and *S. enteritidis*. Contrastingly, the antibiotic ampicillin displayed much higher antibacterial activity against these four pathogens, with MIC values of 1, 2, 8, and 2 μg/ml, respectively. However, compounds **2** and **3** exhibited no activity against the four pathogenic bacteria (data not shown).

### Cytotoxic Activity of Bioactive Compounds

As reported in Table 5, compound **1** displayed excellent cytotoxicity against human lung cancer cells A549 (IC_50_ = 1.00 μg/ml) and human liver cancer cells HepG2 (IC_50_ = 0.91 μg/ml), results which were comparable to that of doxorubicin (IC_50_ = 0.41 and 0.59 μg/ml). In contrast, compound **4** displayed moderate cytotoxicity in the A549 and HepG2 cells with IC_50_ values of 18.95 and 10.67 μg/ml, respectively. Additionally, the IC_50_ of compounds **1** and **4** on normal human primary fibroblast cells WI-38 were 38.07 and 107.35, respectively, thus indicating their selective cytotoxicity against cancer cell lines. The other two compounds showed no obvious cytotoxicity (data not shown).

## Discussion

Over the last few decades, endophytic fungi have been proven to be an untapped resource for novel bioactive compounds [4]. Here, we isolated the endophytic fungi from *L. supina* for further analysis. The whole plant of *L. supina* (Xiazhicao in Chinese) has been described in “Shengnong’s Herbal Classics” and used for over 2,500 years as a traditional Chinese medicine [25]. To our knowledge, this is the first report on the endophytic fungi of *L. supina*.

Nowadays, food loss caused by plant pathogens and infections due to human pathogens is urgent problems to be solved in modern agriculture and modern medicine [26, 27], respectively. As both plant and human pathogens can develop resistance to and reduce their effectiveness of existing drugs, there is an urgent need to develop new antimicrobial drugs. Screening antimicrobial compounds from endophytic fungi has been considered an effective way to overcome the growing antimicrobial resistance of human and plant pathogens [28, 29]. To increase the probability of obtaining active products, all endophytic fungi were preliminarily screened via anti-fungal and antibacterial assays. The XZC-1 strain, which exhibited the best antimicrobial activity was determined as *A. ochraceus* and selected for further study.

*A. ochraceus* is widely distributed in soils and various agricultural commodities and is also known as a food pathogen [30]. However, this fungus can synthesize diverse beneficial metabolites, including polyketides, alkaloids, steroids, peptides, and quinones, with promising bioactivities [31]. For example, three asperochrins with significant antibacterial activity against aquatic pathogenic bacteria were obtained from the marine mangrove-derived fungus *A. ochraceus* [32]. Moreover, three cytotoxic steroid derivatives were discovered from the algal-derived endophytic fungus *A. ochraceus* EN-31 [33]. Additionally, several nitrobenzoyl sesquiterpenoids from the marine alga-derived *A. ochraceus* Jcma1F17 showed excellent cytotoxicity against three renal carcinoma cell lines [34]. Therefore, *A. ochraceus* can produce compounds with interesting structural features and diverse bioactivities, thus making it an attractive target for antimicrobial biosynthesis, chemical synthesis, and bioactivity investigations.

The crude extracts of fungal fermentation products feature a complex composition of multiple compounds. In this case, bioassay-guided fractionation was employed as it connects analytical technique with biological activity and favors the rapid discovery of active antimicrobial compounds [35]. Using bioassay-guided fractionation of the fermented crude extract from *A. ochraceus* XZC-1, we isolated four compounds identified as 2-methoxy-6-methyl-1,4-benzoquinone (**1**), 3,5-dihydroxytoluene (**2**), oleic acid (**3**), and penicillic acid (**4**). Since the crude extract of *A. ochraceus* XZC-1 displayed anti-fungal and antibacterial activity, the four compounds were tested for antimicrobial activity. Among them, only methoxy-6-methyl-1,4-benzoquinone (**1**) and penicillic acid (**4**) were proven to have promising anti-fungal and antibacterial activity. As far as we know, the high in vitro anti-fungal and antibacterial activity of methoxy-6-methyl-1,4-benzoquinone has not been previously reported. However, penicillic acid is a proven anti-fungal agent against the *Phytophthora* species [36]. This study further demonstrated that penicillic acid can also inhibit other phytopathogenic fungi, including *F. species*, *B. cinerea*, *M. oryzae*, and *V. dahliae*. Additionally, penicillic acid was previously reported as an antibacterial agent against various phytopathogenic bacteria, including *Agrobacterium tumefaciens*, *Ralstonia solanacearum*, *Pseudomonas syringae*, and *Xanthomonas* species [37]. Our study provided evidence of its bacteriostatic activity against clinically pathogenic bacteria.

Furthermore, cancer is currently a major cause of death in most countries worldwide [38]. According to the International Agency for Research on Cancer, there were ~10 million deaths from cancer worldwide in 2020 [39]. Endophytic fungi have been reported to be promising producers of bioactive anticancer compounds [40]. Therefore, the compounds were also tested for cytotoxic activity. In our study, methoxy-6-methyl-1,4-benzoquinone and penicillic acid exhibited significant and selective cytotoxicity against the cancer cell lines as compared with the normal fibroblast cells, which was consistent with previous reports [41, 42].

This study reported the isolation and identification of endophytic fungi from *L. supina*. The isolated strain *A. ochraceus* XZC-1 exhibited excellent bioactivity and was further upscale fermented to evaluate its metabolites. Four small molecules, 2-methoxy-6-methyl-1,4-benzoquinone (**1**), 3,5-dihydroxytoluene (**2**), oleic acid (**3**) and penicillic acid (**4**) were obtained and elucidated. Moreover, compounds **1** and **4** showed potent antimicrobial activity against seven phytopathogenic fungi and four human pathogenic bacteria. In addition, these two compounds also displayed promising selective cytotoxicity against human cancer cells (A549 and HepG2). Therefore, based on this study, the endophytic fungi from *L. supina* can be regarded as an important source of antimicrobial and antitumor bioactive compounds.

## Figures and Tables

**Table 1:** Antimicrobial activity of the five endophytic fungal crude extracts.

Endophytic fungi	*F. graminearum*	*S. aureus*

Inhibition zones (mm)		
XZC-1	25	32
XZC-2	20	16
XZC-3	15	24
XZC-4	20	15
XZC-5	15	30

**Table 2:** Antimicrobial activity of the fungal crude extract from XZC-1.

Phytopathogenic fungi	Inhibition rates (%)		Pathogenic bacteria	Inhibition zone diameters (mm)
*F. graminearum*	61.2		*S. aureus*	34
*F. oxysporum*	32.7		*E. coli*	28
*F. moniliforme*	42.3		*L. monocytogenes*	33
*F. stratum*	44.6		*S. enteritidis*	32
*B. cinerea*	55.3			

**Table 3:** MIC values of compounds 1, 4, and actidione against the phytopathogenic fungi.

Pathogenic fungi	Pathogenic fungi (MIC μg/ml)		

	Compound **1** (μg/ml)	Compound **4** (μg/ml)	Actidione (μg/ml)
*F. graminearum*	256	256	128
*F. oxysporum*	256	256	128
*F. moniliforme*	256	256	128
*F. stratum*	256	512	128
*B. cinerea*	256	256	128
*M. grisea*	512	256	64
*V. dahliae*	128	512	64

**Table 4:** MIC values of compounds 1, 4, and ampicillin sodium against pathogenic bacteria.

Pathogenic bacteria	Compound **1** (μg/ml)	Compound **4** (μg/ml)	Ampicillin (μg/ml)
*S. aureus*	8	32	1
*L. monocytogenes*	16	64	2
*E. coli*	64	64	8
*S. enteritidis*	16	64	2

**Table 5:** IC_50_ values of compounds 1, 4, and doxorubicin against cancer cells (A549 and HepG2) and normal human primary fibroblast cells (WI-38).

Cancer cells	A549	HepG2	WI-38

IC_50_ (μg/ml)			
Compound **1**	1.00 ± 0.08	0.91 ± 0.05	38.07 ± 2.25
Compound **4**	18.95 ± 0.55	10.67 ± 1.10	107.35 ± 1.38
Doxorubicin	0.41 ± 0.04	0.59 ± 0.03	12.7 ± 1.38
